# Ethanol Sensitization during Adolescence or Adulthood Induces Different Patterns of Ethanol Consumption without Affecting Ethanol Metabolism

**DOI:** 10.3389/fnbeh.2017.00046

**Published:** 2017-03-23

**Authors:** Priscila F. Carrara-Nascimento, Lucas B. Hoffmann, Marcos B. Contó, Tania Marcourakis, Rosana Camarini

**Affiliations:** ^1^Laboratory of Neurochemistry and Behavioral Pharmacology, Department of Pharmacology, Institute of Biomedical Sciences, Universidade de São PauloSão Paulo, Brazil; ^2^Department of Clinical and Toxicological Analysis, School of Pharmaceutical Sciences, Universidade de São PauloSão Paulo, Brazil

**Keywords:** ethanol, adolescence, behavioral sensitization, voluntary ethanol consumption, aldehyde dehydrogenase, ethanol metabolism

## Abstract

In previous study, we demonstrated that ethanol preexposure may increase ethanol consumption in both adolescent and adult mice, in a two-bottle choice model. We now questioned if ethanol exposure during adolescence results in changes of consumption pattern using a three-bottle choice procedure, considering drinking-in-the-dark and alcohol deprivation effect as strategies for ethanol consumption escalation. We also analyzed aldehyde dehydrogenase (ALDH) activity as a measurement of ethanol metabolism. Adolescent and adult Swiss mice were treated with saline (SAL) or 2.0 g/kg ethanol (EtOH) during 15 days (groups: Adolescent-SAL, Adolescent-EtOH, Adult-SAL and Adult-EtOH). Five days after the last injection, mice were exposed to the three-bottle choice protocol using sucrose fading procedure (4% + sucrose vs. 8%–15% ethanol + sucrose vs. water + sucrose) for 2 h during the dark phase. Sucrose was faded out from 8% to 0%. The protocol was composed of a 6-week acquisition period, followed by four withdrawals and reexposures. Both adolescent and adult mice exhibited ethanol behavioral sensitization, although the magnitude of sensitization in adolescents was lower than in adults. Adolescent-EtOH displayed an escalation of 4% ethanol consumption during acquisition that was not observed in Adult-EtOH. Moreover, Adult-EtOH consumed less 4% ethanol throughout all the experiment and less 15% ethanol in the last reexposure period than Adolescent-EtOH. ALDH activity varied with age, in which older mice showed higher ALDH than younger ones. Ethanol pretreatment or the pattern of consumption did not have influence on ALDH activity. Our data suggest that ethanol pretreatment during adolescence but not adulthood may influence the pattern of ethanol consumption toward an escalation in ethanol consumption at low dose, without exerting an impact on ALDH activity.

## Introduction

Some characteristics of the adolescence (impulsivity, risky behavior, seeking of new experiences) have been related to delayed maturation of prefrontal cortex and neurotransmitter systems as well as late development of behavioral inhibitory systems, which may render adolescents especially vulnerable to taking drugs of abuse and developing addiction (Spear, 2000; Chambers et al., 2003). Ethanol exposure during adolescence can cause dramatic neurobehavioral and neurotoxicological effects compared to exposure during adulthood, as described in humans (Grant and Dawson, 1997; De Wit et al., 2000; Ehlers et al., 2006) and rodents (Crews et al., 2000; Faria et al., 2008; Walker and Ehlers, 2009; Guerri and Pascual, 2010; Soares-Simi et al., 2013; Carrara-Nascimento et al., 2014).

The main route of ethanol elimination is the liver metabolism, where it is converted into acetaldehyde by alcohol dehydrogenase and subsequently to acetate by aldehyde dehydrogenase (ALDH). Those enzymes are responsible for the elimination of alcohol in concentrations below 20 mmol/L (Li, 1977; Lieber, 1986). The efficacy of ethanol metabolism increases with age following its systemic administration, since the blood ethanol concentration remains higher for longer time in younger rats compared to older ones (Kelly et al., 1987). In fact, liver alcohol dehydrogenase efficiency and ALDH activity varies with age (Collins et al., 1975; Hollstedt et al., 1977). Recent data of our group suggested that adult but not adolescent mice developed metabolic tolerance to increases in blood ethanol concentration induced by chronic intermittent ethanol exposure (Carrara-Nascimento et al., 2013), suggesting that the age of exposure to ethanol may also influence ethanol metabolism.

The activity of ALDH may exert some influence on ethanol consumption, since accumulation of acetaldehyde in the peripheral system induces aversive effects when accumulated in the blood (Quertemont, 2004). As an example, high alcohol-drinkers show faster acetaldehyde metabolism and are less vulnerable to its aversive effects, such as flushing, headache, tachycardia, dizziness and nausea (Quintanilla et al., 2006).

Animal models that promote motivation for alcohol seeking/intake include alcohol withdrawal periods since periods of abstinence lead to progressive increases in alcohol consumption that ultimately results in the relief of the abstinence-induced withdrawal symptoms (the so called alcohol deprivation effect—ADE; Spanagel and Hölter, 1999). Drinking in the dark (DID) paradigm is considered a binge-like model since it promotes high levels of blood ethanol concentration (Rhodes et al., 2005). Another procedure to promote increase in ethanol consumption is to preexpose the animals to the drug (Lessov et al., 2001; Camarini and Hodge, 2004; Carrara-Nascimento et al., 2014).

In the present study, we designed a protocol that includes some aspects of human alcohol addiction, such as age of first contact with ethanol, ADE and DID. We hypothesized that mice exposed to ethanol during adolescence would have higher ethanol consumption later in life. We also assessed whether these differences in ethanol consumption pattern might be related to ALDH activity in the liver.

## Materials and Methods

### Animals

Adolescent (PND 28) and adult (PND 68) male Swiss mice were obtained from the Animal Facility of the Department of Pharmacology of the Institute of Biomedical Sciences at the University of São Paulo, Brazil. Mice were housed in groups of five in standard Plexiglas cages (30 cm × 20 cm × 12.5 cm) in a colony room with controlled lighting (12:12 light/dark cycle; lights on from 7:00 AM to 7:00 PM) and temperature (22 ± 2°C) conditions. Mice were allowed to adapt to the colony room for at least 7 days before the start of the experiment. Food and water were provided *ad libitum*. All procedures were approved by the Ethics Committee on Animal Use (Comitê de Ética no Uso de Animais—CEUA—Protocol #18/2013) of the Institute of Biomedical Sciences at the University of São Paulo. Animals were single housed only during the 2-h period of the ethanol consumption procedure.

### Drugs

Ethanol (EtOH, 95% v/v, Merck do Brasil, Rio de Janeiro, Brazil) was diluted in 0.9% w/v sodium chloride (saline, SAL) and injected intraperitoneally (i.p.) as 20% v/v ethanol solution at a dose of 2.0 g/kg during the protocol of behavioral sensitization. Control animals received equivalent volumes of SAL.

For the voluntary ethanol consumption procedure, 95% v/v EtOH was diluted in tap water to produce EtOH solutions according to the concentrations described in Table 1 (4, 8, 10, 12.5 and 15% ethanol v/v).

**Table 1:** **Voluntary ethanol consumption**.

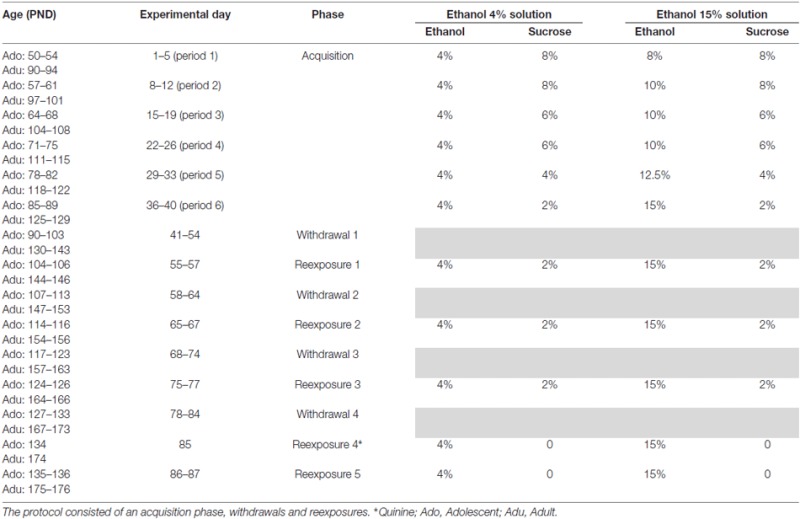

### Experimental Design

**Figure 1:**
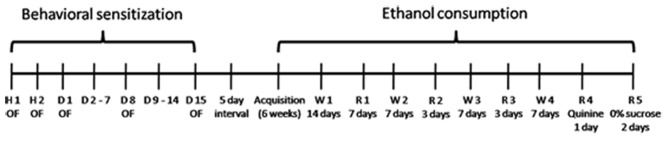
**Behavioral sensitization**: on Habituation days 1 and 2 (H1 and H2) mice were treated with saline (SAL). From treatment days 1–15 (D1–D15) mice received intraperitoneal (i.p.) injections of SAL or 2 g/kg Ethanol. OF: locomotor activity assessment in the open-field. **Ethanol consumption**: 5 days after behavioral sensitization procedure, mice were exposed to voluntary ethanol consumption protocol, which consisted of an acquisition phase, followed by withdrawals and reexposures to the three-bottle choice (water, 4% ethanol and 15% ethanol).

The whole experimental design is shown in Figure 1. It involves two phases: Phase 1 (Behavioral Sensitization) and Phase 2 (Voluntary Ethanol Consumption).

### Phase 1—Behavioral Sensitization

Fifty-seven mice were used for this experiment. Their locomotor activity was assessed using a cylindrical wooden-made open-field arena (40 cm diameter and 35 cm height). A video camera placed above the apparatus and connected to a computer located outside the experimental room recorded the trials. Five minutes after SAL or EtOH injections, the animals’ locomotor activity (distance traveled in cm) was assessed during 5 min and quantified with Ethovision software (Noldus, Wageningen, Netherlands). The 5-min trial duration after 5 min of ethanol injection is based on previous pilot studies conducted in our laboratory and on studies showing optimal ethanol sensitization between 5 and 10 min after injection (Broadbent and Harless, 1999; Meyer and Phillips, 2007). The apparatus was cleaned with a 5% ethanol/water solution between each trial.

In order to let the animals to habituate to the injection procedure and the open-field apparatus, mice were first injected with SAL for two consecutive days prior to the treatment with EtOH. Adolescent and adult mice received an injection of SAL and were placed in the open-field apparatus to assess their locomotor activity. From the next day on, mice were treated for 15 consecutive days with i.p. injections of SAL or 2.0 g/kg of EtOH once a day. Therefore, there were four experimental groups: Adolescent-SAL (*n* = 15), Adolescent-EtOH (*n* = 14), Adult-SAL (*n* = 14) and Adult-EtOH (*n* = 14). Group names refer to age and treatment in which mice received SAL or EtOH to induce behavioral sensitization during adolescence or adulthood. It is important to emphasize that mice were preexposed i.p. to ethanol during adolescence or adulthood and the testing (consumption) was actually performed during either adulthood or young adulthood. By using this protocol, we aimed to assess ethanol consumption in adult mice preexposed to ethanol during adolescence. Locomotor activity was assessed on days 1, 8 and 15. Injections and locomotor activity assessment were always carried out between 9:00 AM and 11:30 AM. Following this first phase of the protocol, mice underwent 5 days of abstinence before being exposed to the voluntary ethanol consumption.

### Phase 2—Voluntary Ethanol Consumption—Drinking in the Dark

The protocol of this phase is shown in Table 1.

During the Phase 1 of the experiment, one mouse from Adult-SAL and one from Adult-EtOH died.

Three hours after the lights were turned off (9:00 AM), animals had access to three-bottle choice: one water bottle and two bottles containing different ethanol concentrations, for 2 h according to the DID procedure (Rhodes et al., 2005; Crabbe et al., 2014). The voluntary ethanol consumption consisted of: *Acquisition*: we reiterate that animals belonging to Adolescent groups reached post-adolescence period during Phase 2 of this study. We used a modified sucrose fading procedure (Samson, 1986) because Swiss mice are not classified as high preferring mice. Instead, this mouse strain shows high variability of ethanol drinking patterns (Ribeiro et al., 2012). The sucrose fading procedure is used when the taste aversion to ethanol may be a problem in initiation of drinking. During acquisition phase, mice were exposed to three-bottle choice for five consecutive days followed by 2 days of abstinence. This procedure was repeated six times. The short withdrawals were included to accelerate the ethanol intake. The sucrose concentration in both ethanol bottles was gradually reduced from 8% to 2%. The ethanol concentration in one of the bottles was of 4% throughout the whole experiment, while the ethanol concentration in the other bottle was gradually increased from 8% to 15%. *Withdrawals and reexposures*: four withdrawal periods were intercalated with five reexposures. The first withdrawal lasted 14 days whilst the others lasted 7 days. This protocol was based on studies showing that a longer withdrawal may result in increased ethanol consumption in the following reexposures (Rodd-Henricks et al., 2000, 2001; Rodd et al., 2003, 2009). Each of the three first withdrawals was followed by a reexposure period (resulting in Reexposures 1, 2 and 3). Each reexposure period consisted of 2-h access to ethanol/day for three consecutive days. During these reexposures mice had access to the three-bottle choice (4% ethanol + 2% sucrose, 15% ethanol + 2% sucrose and water). On the fourth reexposure (2-h access to ethanol for 1 day) ethanol solutions were adulterated with 0.005 g/L quinine and no sucrose was added. The quinine concentration was chosen based on previous studies showing that this concentration in water creates an aversive bitter taste and reduces its intake without causing total inhibition of intake (Fachin-Scheit et al., 2006; Vendruscolo et al., 2012; Leão et al., 2015). On the next day, during the fifth reexposure (2-h access to ethanol/day for two consecutive days) the sucrose was completely faded and no quinine was added to the ethanol solutions.

The ethanol intake data from each set of days within each period of exposure of the three-bottle choice protocol was averaged and plotted as a single time point in the graph.

Ethanol intake was calculated in grams per kilogram of mice body weight (g/kg) according to the formula:

volume consumed(mL) ​×ethanol concentration in the solution×ethanol density(g/mL)​/mouse body weight(kg).

### Quantification of Aldehyde Dehydrogenase (ALDH) Activity

Immediately after the last reexposure, mice were euthanized by cervical dislocation. The livers were collected, immediately frozen and kept at −80°C. Eight mice from each group were randomly chosen for enzyme analysis.

The ALDH assay was performed according to the description of the manufacturer (GWB-AXR339, Genway). Briefly, liver tissues (50 mg) were homogenized with 200 μL of ice cold buffer. The homogenates were left for 10 min on ice, centrifuged at 12,000 g for 5 min at 4°C to remove nuclei and insoluble material and the resulting supernatants were collected to be used in the assay. The principle of the colorimetric assay kit consists in the oxidation of acetaldehyde by the enzyme ALDH of the sample. The reaction generates NADH that reduces an uncolored probe into a colored product with strong absorbance at 450 nm. The samples were read in a spectrophotometer at a wavelength of 450 nm in a kinetic mode (each 2 min), picking the linear range within NADH standard curve. The activity of ALDH was determined by subtracting the values in the absence of the substrate acetaldehyde from the values in the presence of the substrate (performed in duplicates). A standard curve was performed using five distinct amounts of NADH ranging from 2 nmol to 10 nmol, and the ALDH activity was calculated as nmol of NADH released/min/mL.

### Statistical Analysis

The behavioral sensitization data was analyzed with a two-way ANOVA (habituation: age × days) and three-way ANOVA (treatment: age × days × treatment) and days were used as repeated measure.

The ethanol consumption data was analyzed using a three-way ANOVA (age × treatment × time) with time as repeated measure. When necessary, three-way ANOVA for repeated measures was deconstructed into two-way ANOVAs (age × time) to evaluate age differences within each treatment. A two-way ANOVA (age × treatment) was performed to analyze reexposure 4 (quinine adulteration) and reexposure 5 (0% sucrose).

Data from the ALDH activity was analyzed using a two-way ANOVA (age × treatment).

Newman-Keuls was used for all *post hoc* comparisons.

For all analysis performed, statistical significance was considered when *p* < 0.05. We used the program STATISTICA 7 (StatSoft) to analyze the data.

## Results

### Behavioral Sensitization

#### Habituation

A two-way ANOVA (age × days) for repeated measures revealed an effect of time. Locomotor activity decreased in the second day compared to the first day (*F*_(1,55)_ = 37.66; *p* < 0.01), showing habituation to the apparatus.

#### Repeated Ethanol Treatment

**Figure 2:**
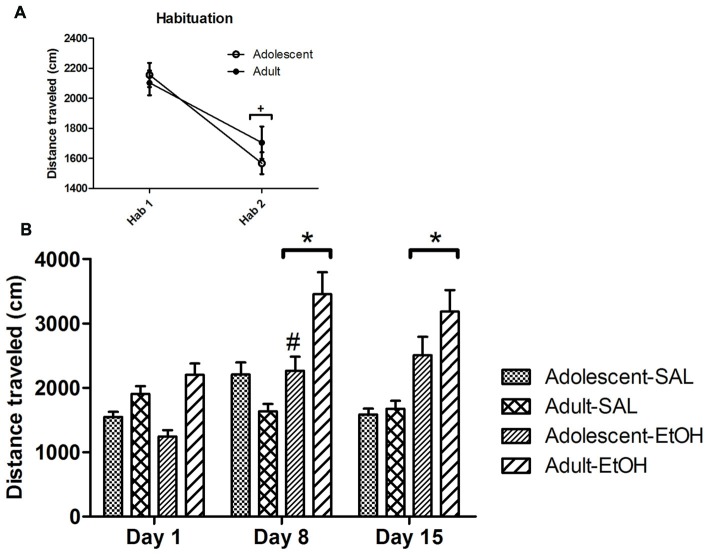
**Behavioral sensitization.** The main graph **(B)** illustrates the locomotor activity of adolescent and adult mice repeatedly treated with i.p. injections of SAL or 2.0 g/kg ethanol (EtOH) during 15 consecutive days (Adolescent-SAL, *n* = 15; Adult-SAL, *n* = 14; Adolescent-EtOH, *n* = 14; Adult-EtOH, *n* = 14). The locomotor activity was assessed on Days 1, 8 and 15. The smaller graph **(A)** illustrates locomotor activity on Habituation Days 1 and 2, when all mice received SAL injections. ^+^H2 < H1; *Locomotor activity was higher than on Day 1; ^#^Adolescent-EtOH < Adult EtOH.

A three-way ANOVA (age × treatment × days) revealed effects of age (*F*_(1,53)_ = 8.96, *p* < 0.01), treatment (*F*_(1,53)_ = 22.67, *p* < 0.01), age × treatment (*F*_(1,53)_ = 10.71, *p* < 0.01), days (*F*_(2,106)_ = 18.81, *p* < 0.01), treatment × days (*F*_(2,106)_ = 15.72, *p* < 0.01) and age × treatment × days interaction (*F*_(2,106)_ = 4.35, *p* < 0.05). Mean comparisons among treatments showed that mice treated with ethanol displayed greater locomotor activity than those treated with SAL. *Post hoc* analysis of the significant age × treatment effect revealed that the locomotor activity in Adolescent-EtOH was lower than in Adult-EtOH mice. Pairwise comparisons of the significant age × treatment × days interaction showed that both adolescent and adult mice treated with ethanol displayed higher locomotor activity on days 8 and 15 as compared to day 1, revealing that they developed behavioral sensitization. On day 8, Adolescent-EtOH showed a lower locomotor activity than Adult-EtOH. The locomotor activity of all groups on Day 1 was analyzed by a two-way ANOVA (age × treatment) and revealed a tendency to hypolocomotor activity after an acute ethanol injection in adolescent mice (*p* = 0.08), while adult mice showed the opposite effect (*p* = 0.09; Figure 2).

### Voluntary Ethanol Consumption

**Figure 3:**
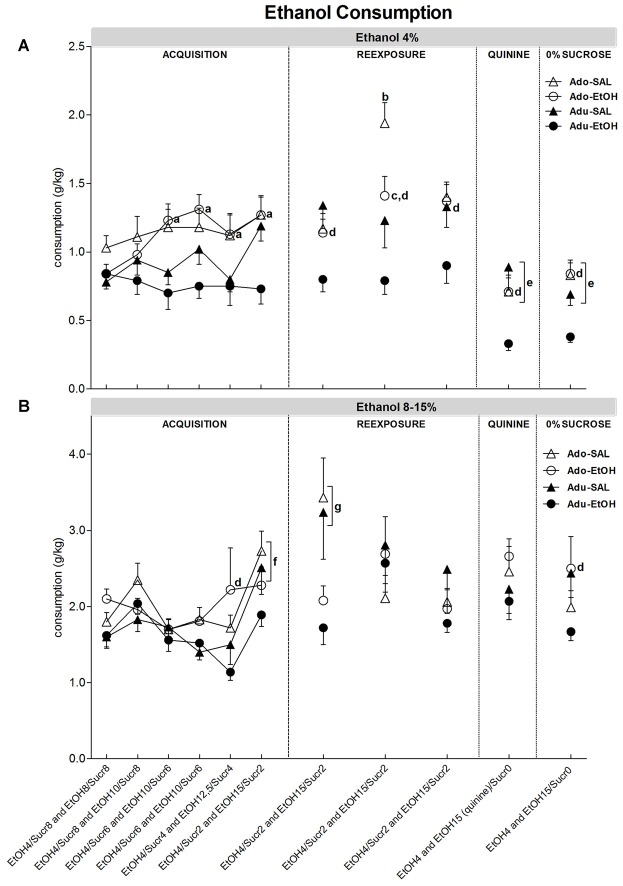
**Voluntary ethanol consumption.** The figure contains graphs illustrating ethanol consumption during acquisition and reexposure periods to 4% EtOH solution **(A)** and 8%–15% EtOH solution **(B)**, as described in Table 1. Animals previously treated during adolescence or adulthood with SAL or EtOH were exposed to the three-bottle choice protocol: water vs. 4% EtOH vs. 8%–15% EtOH. The *x* axis shows the concentration of ethanol and sucrose in each ethanol bottle within each consumption period. The whole experimental protocol of this phase is described in Table 1. The letters indicate the following statistically significant differences: a = Adolescent-EtOH displayed greater 4% ethanol intake on acquisition periods 3, 4, 5 and 6 compared to period 1; b = Adolescent-SAL displayed greater 4% ethanol consumption on reexposure 2 compared to reexposures 1 and 3; c = Adolescent-EtOH displayed greater 4% ethanol consumption on reexposure 2 compared to reexposure 1; d = Adolescent-EtOH consumed more ethanol than Adult-EtOH; e = Adult and Adolescent groups exhibited reduced ethanol consumption on the reexposures 4 and 5 as compared to the previous periods (acquisition 6, reexposures 1, 2 and 3); f = Ethanol intake on period 6 was higher than on period 1; g = SAL groups showed higher EtOH intake than EtOH groups on reexposure1 (Adolescent-SAL, *n* = 15; Adult-SAL, *n* = 14; Adolescent-EtOH, *n* = 13; Adult-EtOH, *n* = 13).

The results are shown in Figure 3.

### Ethanol 4% (Figure 3A)

We first performed a repeated three-way ANOVA (age × treatment × time) considering the last period of acquisition phase (when ethanol consumption was stabilized) and reexposures (1–5) as repeated measures. There were significant effects of age (*F*_(1,51)_ = 10.85, *p* < 0.01), treatment (*F*_(1,51)_ = 9.36, *p* < 0.01), age × treatment (*F*_(1,51)_ = 4.44, *p* < 0.05), time (*F*_(5,255)_ = 37.12, *p* < 0.01), age × time (*F*_(5,255)_ = 4.85, *p* < 0.01), but no age × treatment × time interaction. The age × treatment interaction effect showed that Adult-EtOH consumed less ethanol than the other groups (Adolescent-EtOH, Adolescent-SAL, Adult-SAL; *F*_(1,52)_ = 4.35, *p* < 0.05). The age × time interaction effect (*F*_(5,255)_ = 4.85, *p* < 0.01) demonstrated that Adult groups and Adolescent groups exhibited reduced ethanol consumption on the reexposures 4 (quinine adulteration) and 5 (0% sucrose) as compared to the previous periods (acquisition 6, reexposures 1, 2 and 3).

Following this analysis, we performed ANOVAs for each of the phases of ethanol consumption.

#### Acquisition

A three-way ANOVA for repeated measures revealed effects of age (*F*_(1,51)_ = 10.74, *p* < 0.01), time (*F*_(5,255)_ = 4.51, *p* < 0.01) and age × treatment × time interaction (*F*_(5,255)_ = 2.66, *p* < 0.05). A two-way ANOVA performed to analyze SAL groups revealed no age × time interaction. A two-way ANOVA performed to analyze EtOH groups revealed effects of age (*F*_(1,25)_ = 10.64, *p* < 0.01) and age × time interaction (*F*_(5,125)_ = 3.99, *p* < 0.01). *Post hoc* analysis showed that Adolescent-EtOH exhibited higher ethanol consumption compared to Adult-EtOH. Adolescent-EtOH mice but not Adult-EtOH showed escalation of 4% ethanol intake, since ethanol intake was greater on acquisition periods 3, 4, 5 and 6 compared to first acquisition period in the Adolescent-EtOH.

#### Reexposures

A three-way ANOVA for repeated measures used to evaluate reexposures 1, 2 and 3 revealed effects of age (*F*_(1,51)_ = 9.47, *p* < 0.01), treatment (*F*_(1,51)_ = 8.2, *p* < 0.01), time (*F*_(2,102)_ = 6.35, *p* < 0.01), age × time interaction (*F*_(2,102)_ = 10.14, *p* < 0.01) and age × treatment × time interaction (*F*_(2,102)_ = 3.1, *p* < 0.05). Adult-EtOH mice displayed lower ethanol intake compared to the other groups. The three-way ANOVAs were deconstructed into two-way ANOVAs to evaluate age differences within each treatment. A two-way ANOVA used to analyze the SAL groups revealed effects of time (*F*_(2,52)_ = 4.05, *p* < 0.05) and age × time interaction (*F*_(2,52)_ = 7.55, *p* < 0.01). Adolescent-SAL displayed greater 4% ethanol consumption on reexposure 2 compared to reexposures 1 and 3. A two-way ANOVA performed to analyze EtOH groups revealed effects of age (*F*_(1,25)_ = 10.22, *p* < 0.01), time (*F*_(2,50)_ = 5.56, *p* < 0.05) and age × time interaction (*F*_(2,50)_ = 3.43, *p* < 0.05). Adolescent-EtOH displayed greater 4% ethanol intake on reexposure 2 compared to reexposure 1. *Post hoc* analysis of the significant age effect revealed that Adolescent-EtOH exhibited higher ethanol intake than Adult-EtOH mice. A two-way ANOVA performed for reexposure 4 (quinine adulteration) revealed effects of treatment (*F*_(1,51)_ = 5.69, *p* < 0.05) and age × treatment interaction (*F*_(1,51)_ = 5.26, *p* < 0.05). *Post hoc* analysis showed that Adult-EtOH drank less ethanol than Adolescent-EtOH and its respective control group (Adult-SAL), and almost reached statistical significance compared to Adolescent-SAL (*p* = 0.07). Analysis of reexposure 5 (0% sucrose) by a two-way ANOVA (age × treatment) revealed that Adult-EtOH mice displayed lower ethanol intake compared to the other groups (*F*_(1,52)_ = 4.25, *p* < 0.05).

### Ethanol 8%–15% (Figure 3B)

A repeated three-way ANOVA was performed considering the last period of acquisition phase and reexposures as repeated measures. A significant effect of treatment × time was found (*F*_(5,255)_ = 4.23, *p* < 0.05).

#### Acquisition

A three-way ANOVA for repeated measures revealed effects of age (*F*_(1,51)_ = 5.19, *p* < 0.05), time (*F*_(5,255)_ = 9.93, *p* < 0.01) and treatment × time interaction (*F*_(5,255)_ = 2.32, *p* < 0.05). No age × treatment × time interaction was found. A two-way ANOVA performed to analyze SAL groups revealed an effect of time (*F*_(5,130)_ = 16.67, *p* < 0.05). A two-way ANOVA performed to analyze EtOH groups revealed an effect of age (*F*_(1,25)_ = 4.46, *p* < 0.05). Although we have found a statistically significant age effect (Adolescent-EtOH drank more ethanol than Adult-EtOH), the difference comes only from the acquisition period 5. *Post hoc* analysis of the significant time effect showed that ethanol intake on acquisition period 6 was higher than on acquisition period 1 for both adolescent and adult SAL groups.

#### Reexposures

A three-way ANOVA for repeated measures used to analyze reexposures 1, 2 and 3 revealed effects of treatment (*F*_(1,51)_ = 4.72, *p* < 0.05) and time × treatment interaction (*F*_(2,102)_ = 5.49, *p* < 0.01). *Post hoc* analysis of the significant time × treatment effect showed that SAL groups drank more ethanol than EtOH groups on reexposure 1. SAL groups also showed a gradual decrease in ethanol intake over time. A two-way ANOVA used to analyze reexposure 4 (quinine adulteration) revealed no significant effect. A two-way ANOVA used to analyze reexposure 5 (0% sucrose) revealed an age × treatment interaction, in which Adult-EtOH drank less ethanol than Adolescent-EtOH (*F*_(1,51)_ = 5.26, *p* < 0.05).

### ALDH Activity

**Figure 4:**
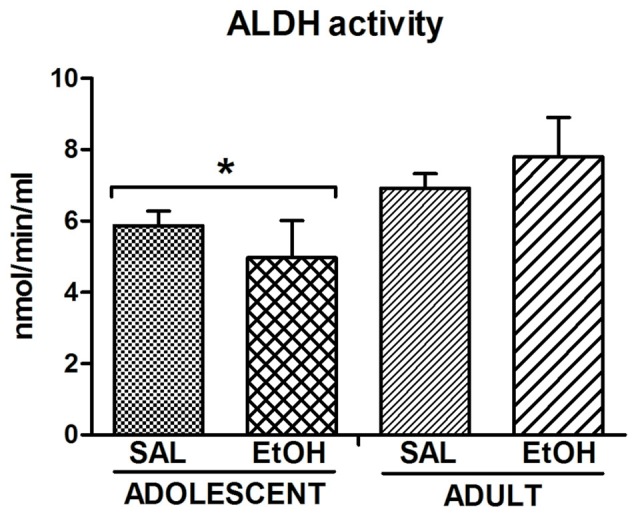
**Aldehyde dehydrogenase (ALDH) activity.** The enzyme activity was measured in the liver of mice (*n* = 8 mice/group) previously treated with repeated i.p. injections of SAL or EtOH during 15 days and subsequently exposed to a voluntary ethanol consumption protocol that consisted of an acquisition phase followed by withdrawals and reexposures (Table 1). *Decreased enzymatic activity compared to Adults.

Data from ALDH activity was analyzed using a two-way ANOVA (age × treatment), which showed an effect of age (*F*_(1,28)_ = 4.66, *p* < 0.05). *Post hoc* analysis showed that Adolescent groups (-SAL and -EtOH) exhibited lower ALDH activity as compared to Adult groups (-SAL and -EtOH; Figure 4).

## Discussion

The current study proposes an experimental protocol that includes risk factors for addiction (adolescence period), incentive salience (behavioral sensitization) and induction of binge-like consumption (withdrawal and reexposures during dark period) to resemble some of the aspects of the addiction in humans. We showed that both adolescent and adult mice treated with 2.0 g/kg ethanol (Adolescent-EtOH and Adult-EtOH) displayed ethanol behavioral sensitization and that adolescents were less sensitive than adults, which is in agreement with studies from our laboratory and others (Stevenson et al., 2008; Quoilin et al., 2012; Soares-Simi et al., 2013; Carrara-Nascimento et al., 2014). The most striking result is that Adolescent-EtOH but not Adult-EtOH displayed escalated amounts of 4% ethanol intake during acquisition and maintained higher ethanol intake than Adult-EtOH after repeated withdrawals and reexposures, even when ethanol solution was adulterated with quinine or when sucrose was reduced to 0%. Age-related differences in 15% ethanol intake emerged only during the last reexposure (reexposure 6) after repeated withdrawals. In all phases, Adult-EtOH mice displayed lower 4% ethanol intake compared to the other groups.

Repeated cycles of withdrawals and reexposures have been used to increase ethanol consumption. In our previous study (Carrara-Nascimento et al., 2014), using behavioral sensitization paradigm as pretreatment exposure, we demonstrated that ethanol pretreated mice showed higher 10% ethanol intake when compared to SAL pretreated mice, regardless of age of preexposure or behavioral sensitization magnitude. Adolescent and adult mice exposed to chronic ethanol vapor chamber also increased ethanol intake similarly in a two-bottle choice test (Carrara-Nascimento et al., 2013).

In the current study, using three-bottle choice test, age-differences in ethanol-pretreated mice emerged when sucrose concentration was 6% in the 4% ethanol solution, in that Adolescent-EtOH consumed more sweetened 4% ethanol solution than Adult-EtOH. We could explain the gradual divergence between those groups based on the facts that: (1) low ethanol concentration may be perceived as more palatable than high concentration; (2) adolescent animals consume more sucrose than adults; (Anderson et al., 2010); and (3) adolescents present higher sensitivity to the hedonic properties of sucrose than adults (Wilmouth and Spear, 2009) and thus, they would increase their consumption because of the appetitive taste of both ethanol and sucrose. However, these explanations do not take in consideration the lack of difference in ethanol intake between Adolescent-SAL and Adult-SAL. Moreover, Maldonado et al. (2008) demonstrated that adolescent rats consumed more ethanol than adults using sweetened alcohol solutions and concluded that sucrose was not relevant to the age difference found. In the present study, the age differences in ethanol pretreated mice were maintained even when sucrose was completely faded out, suggesting that the behavioral sensitization during adolescence or adulthood may account for the age-differences in voluntary ethanol consumption. We may suggest that previous behavioral sensitization decreased ethanol intake in adult but not in adolescent mice. This might be because of the ontogeny of the dopaminergic system with an inverted U-shaped format in brain regions involved in motivation and rewarding (McCutcheon and Marinelli, 2009). Functional characteristics of the dopaminergic system during development have been implicated in distinct patterns of behavioral response to drugs between younger and older animals, such as sensitization and/or consumption (Doremus et al., 2005; Frantz et al., 2007; Camarini et al., 2008; Faria et al., 2008; Valzachi et al., 2013; Camarini and Pautassi, 2016).

Although we have not found differences to the acute simulant effects of ethanol between adolescent and adult mice in the present and previous studies (Faria et al., 2008; Carrara-Nascimento et al., 2011, 2013; Soares-Simi et al., 2013), adolescents showed lower levels of locomotor sensitization to ethanol than adults when receiving low doses of ethanol (Faria et al., 2008; Stevenson et al., 2008). Moreover, it has been demonstrated that female adolescent mice need higher ethanol doses (i.e., 4.0 g/kg) than adults to develop ethanol locomotor sensitization (Quoilin et al., 2012), suggesting ontogenic differences in ethanol-induced behavioral sensitization. However, we cannot discard the hypothesis that the lower activity in adolescents compared to adults is in fact a process of tolerance to the hypolocomotor (sedative) effect of ethanol rather than locomotor sensitization. Although Phillips et al. (1996) have shown that sensitization does not result from tolerance to the sedative effects of ethanol in BXD/Ty recombinant inbred strains, this hypothesis should be further investigated in adolescent mice.

In the present study, sensitized adolescent mice displayed an ethanol consumption pattern that differed from the sensitized adult mice, in that they showed a gradual increase in consumption of 4% ethanol solution. It seems to have an interaction between low sensitivity to behavioral sensitization and consumption of ethanol solution at low concentration. In other words, sensitized adolescent mice drunk ethanol at low concentrations in stimulant doses to reach the appetitive effects of ethanol. The availability of ethanol solution at low concentration allowed those mice to control the ethanol self-administration to reach those effects. Taking this into account, it is likely that the low predisposition to behavioral sensitization in adolescents, in fact, yield animals more prone to escalate alcohol consumption at low concentration.

It has been demonstrated that adolescent rodents are not efficient to titrate their ethanol consumption as adults (Maldonado et al., 2008). Rodents learn to titrate ethanol intake based on their previous experiences with ethanol, likely mediated by postingestional effects of ethanol (Samson et al., 2002; Czachowski et al., 2006). In the present study, preexposure to ethanol during adolescence or adulthood differentially impacted the ability of animals to titrate their ethanol consumption, in that Adult-EtOH consumed less ethanol than Adolescent-EtOH. The statistical analysis that considered all experimental phases revealed that Adult-EtOH displayed lower 4% ethanol intake compared to all the other groups. Moreover, Adult-EtOH mice also drank less 15% ethanol than Adolescent-EtOH during the last phase of the experiment (0% sucrose). Thus, we suggest that previous behavioral sensitization in adult but not in adolescent mice exerted a protective effect in adult mice towards increased ethanol intake in a model of three-bottle choice using sucrose fading procedure. Using a different protocol, we have demonstrated that preexposure to ethanol increased ethanol intake, regardless of age (Carrara-Nascimento et al., 2014). It is noteworthy that in the latter study, the protocol included only one abstinence phase.

A hypothesis to explain the steady escalation of 4% ethanol solution is through pharmacological sensitization (Zernig et al., 2007). It is likely that those mice showed a rapid escalation to reach a state of greater sensitivity to ethanol-induced sensitization, and desired stimulation levels. They also showed persisted ethanol intake throughout the five reexposure periods and higher consumption than Adult-EtOH, confirming the important role of ethanol exposure during adolescence to induce use disorders later on adulthood. It is interesting to note that when more chronic ethanol reexposures were introduced, a significant higher increase in 15% ethanol intake in Adolescent-EtOH group compared to Adult-EtOH group also appeared, suggesting an influence of high ethanol consumption at higher concentrations in the timeline of ethanol exposure. Rodent preferences usually shift to the highest ethanol concentrations after withdrawals in three-bottle choice tests with multiple ethanol concentrations (Hölter et al., 1998; Rodd-Henricks et al., 2001; Ribeiro et al., 2012).

Even though quinine has decreased 4% ethanol consumption in both Adolescent-EtOH and Adult-EtOH mice, the age-differences were maintained, showing that motivation levels to drink were equally preserved. Interestingly, aversion to quinine taste was minimized in 15% ethanol solution. This suggests a great motivation to drink even under aversive taste or, alternatively, quinine bitter tasting was masked by the high ethanol concentration.

ADE exerted a greater effect in younger mice, since consumption of 4% ethanol increased during reexposure 2 in Adolescent-EtOH and Adolescent SAL, which was tolerated in Adolescent-SAL but not in Adolescent-EtOH. Tolerance was also observed to 15% ethanol consumption in both Adolescent-SAL and Adult-SAL. A marked difference between mice pretreated with ethanol or SAL is that non-pretreated mice, regardless of age, showed increased 15% ethanol intake during the last acquisition period and developed tolerance during subsequent reexposures. Moreover, the initial longer ADE induced a greater effect on 15% ethanol consumption in non-pretreated mice compared to sensitized mice (Adolescent-EtOH and Adult-EtOH). Although speculative, these data suggest that behavioral sensitization is not necessarily related to increased ethanol consumption. Discordant results have been reported on the correlation between behavioral sensitization and ethanol consumption. Abrahao et al. (2013) demonstrated an association between locomotor sensitization and ethanol drinking in Swiss mice. The difference between our study and theirs is the number of ethanol bottles during the test, ethanol concentration, and more important, the classification of mice receiving ethanol into “sensitized” and “nonsensitized” in their study. Other important difference in this study from ours is that during the initial phase of the self-administration protocol, animals were given forced exposure to the ethanol solution before having access to the two-bottle choice (Abrahao et al., 2013). Ribeiro et al. (2008) did not find a correlation between these two parameters. Fabio et al. (2014) showed an enhancement of ethanol consumption in adolescent, but not in adult mice, preexposed to binge ethanol intoxication, regardless development of behavioral sensitization.

Regardless of the previous treatment, we found a significant effect of age on ALDH activity, with older mice showing higher activity compared to younger ones, suggesting decreased rate of alcohol metabolism in younger mice. In support of our results, Collins et al. (1975) demonstrated age-differences in ALDH activity between mice from PND = 50–60 and PND = 95–110. We also found that exposure to ethanol during adolescence did not alter ALDH activity on adulthood, since there were no significant differences in ALDH activity between Adolescent-EtOH and Adolescent-SAL. This is a caveat in our study because all mice were exposed to ethanol during voluntary consumption. Although we lack this control, ethanol self-administration studies in rats reported no differences in the ALDH activity between those that were given ethanol compared to their controls (Amir, 1978). In humans, chronic exposure to alcohol increases acetaldehyde in the blood and decreases ALDH activity in the liver (Jenkins and Peters, 1980; Palmer and Jenkins, 1982). Interestingly, reduction in this enzyme activity is related to liver damage or excessive alcohol consumption. Aldehydes have an important role on cell signaling for apoptosis and in the pathophysiology of alcoholism (Kruman et al., 1997; Hayes et al., 2000).

In conclusion, preexposure to ethanol during adolescence may have altered ethanol-induced stimulation threshold. Behavioral sensitization during adolescence or adulthood induced different patterns of ethanol consumption, in that adult but not adolescent preexposed mice showed lower ethanol consumption, without affecting ALDH activity.

## Author Contributions

PFC-N and RC conceived the experiments. PFC-N and LBH conducted the behavioral experiments. PFC-N and MBC performed the aldehyde dehydrogenase activity assay. PFC-N, LBH, TM and RC analyzed the data and participated in drafting the article. RC is responsible for funding and revising the final version.

## Funding

The authors thank Fundação de Amparo à Pesquisa do Estado de São Paulo (FAPESP; São Paulo Research Foundation) for the research funding (grant # 2012/10260-7) and for scholarship to PFC-N (grant # 2012/17228-1) and CNPq (National Council for Scientific and Technological Development) for the research funding (grant # 470070/2012-9). RC and TM are research fellows of CNPq.

## Conflict of Interest Statement

The authors declare that the research was conducted in the absence of any commercial or financial relationships that could be construed as a potential conflict of interest.
